# Draft Genome Sequence of the Coprinoid Mushroom *Coprinopsis strossmayeri*

**DOI:** 10.1128/genomeA.00044-17

**Published:** 2017-04-06

**Authors:** Alice M. Banks, Gary L. A. Barker, Andy M. Bailey, Gary D. Foster

**Affiliations:** School of Biological Sciences, University of Bristol, Bristol, United Kingdom

## Abstract

*Coprinopsis strossmayeri* is a coprinoid mushroom favoring the habitat of herbivore dung. As a result of this highly competitive environment, *C. strossmayeri* is anticipated to produce a wide array of antimicrobial secondary metabolites (SMs) of potential pharmaceutical importance. Here, we present the draft genome sequence of *C. strossmayeri*.

## GENOME ANNOUNCEMENT

Basidiomycete fungi are part of a hugely varied phylum of organisms capable of carrying out a diversity of important roles (1). The coprinoid mushrooms encompass fungi from the *Coprinus*, *Coprinopsis*, and *Coprinellus* genera, and, along with the *Parasola*, comprise the Psathyrellaceae family (2). Most coprinoids favor a habitat of herbivore dung, but growth on decaying woodland material is also common (3). A likely result of residing in such competitive environments and cohabiting with predatory microorganisms is the production of a plethora of bioactive secondary metabolites (SMs) used in defense, many of which can potentially be exploited for human use (4). Terpenoids are the most abundant class of compounds produced by basidiomycetes (5), the derivatives of which have been developed for medical applications, such as the diterpene antibiotic pleuromutilin from *Clitopilus passeckerianus* (6).

The strain sequenced was obtained from the CBS Fungal Biodiversity Centre, submitted as *Coprinus quadrifidus* CBS 177.39. The internal transcribed spacer region was amplified and sequenced to confirm species identity. Subsequent analysis by BLASTn search identified the isolate as *Coprinopsis strossmayeri*, showing 98% identity to *C. strossmayeri* strain SZMC-NL-0774 (GenBank accession number HQ847048.1).

The genomic DNA of dikaryotic *C. strossmayeri* (CBS 177.39) mycelium was sequenced using the Illumina HiSeq 2500 system. Paired-end 100-bp fragments were prepared and sequenced, generating 93,872,638 reads covering a total 9,387 Mbp (≥Q30 bases, 91.87%). Data were processed using RTA version 1.17.21.3, with default settings, and reads were demultiplexed, allowing no mismatches, with CASAVA 1.8.2. Quality-trimmed reads were assembled using CLC Genomics Workbench 6. This resulted in a genome assembly comprising 622 contigs totaling 33,316,483 bp, with an average contig length of 53,563 bp (largest contig, 1,580,550 bp; smallest contig, 1,000 bp). An *N*_25_ contig length of 380,611 bp, *N*_50_ of 190,582 bp, and *N*_75_ of 80,746 bp were obtained. The G+C content was 49%. This genome size is comparable to those of related coprinoid fungi.

Genome analysis was performed using antiSMASH (7). This located five terpene synthase genes related to terpenoid biosynthesis, four of which are characteristic of sesquiterpene biosynthesis, while the other is expected to be involved with diterpene biosynthesis, based on phylogenetic analyses. A local BLAST search of the genome located two geranylgeranyl diphosphate synthases likely to be involved with diterpene biosynthesis. Terpenoid production is well documented among coprinoid fungi; many products of this chemical class have been reported from related species (8–11). One type I partially reducing polyketide synthase and one nonribosomal peptide synthase were also identified through antiSMASH. Interestingly, a locus showing similarity to copsin, an antimicrobial peptide from *Coprinopsis cinerea*, was located through a BLAST search (12). We plan to identify the biosynthetic gene clusters in *C. strossmayeri* responsible for the production of novel antimicrobial SMs and to characterize the genes constituting these pathways. The numbers of genes and gene clusters identified highlight the potential of *C. strossmayeri* as a source of novel terpenoid SMs to be exploited in the pharmaceutical industry.

### Accession number(s).

This whole-genome shotgun project has been deposited in DDBJ/ENA/GenBank under the accession numbers FTPT01000001 to FTPT01000622.
